# CDK4/6抑制剂在非小细胞肺癌治疗中的研究进展

**DOI:** 10.3779/j.issn.1009-3419.2020.03.07

**Published:** 2020-03-20

**Authors:** 琼 秦, 尧尧 任, 殿胜 钟

**Affiliations:** 300052 天津，天津医科大学总医院肿瘤内科 Department of Medical Oncology, Tianjin Medical University General Hospital, Tianjin 300052, China

**Keywords:** 肺肿瘤, 细胞周期蛋白D, CDK4/6抑制剂, 生物标记物, Lung neoplasms, Cyclin D, CDK4/6 inhibitors, Biomarkers

## Abstract

肺癌是全球癌症相关死亡的首要原因，非小细胞肺癌（non-small cell lung cancer, NSCLC）占肺癌的80%-85%。恶性肿瘤可以无限增殖，细胞周期失调控是恶性肿瘤特征之一。细胞周期依赖激酶（cyclin D-dependent kinase, CDK）4/6抑制剂能阻滞肿瘤细胞通过G1期进入S期从而抑制肿瘤增殖，该药物在激素受体阳性乳腺癌中取得了良好的疗效，联合内分泌治疗成为这类患者一线标准治疗。细胞周期失控在NSCLC中比较常见，发生率约为22%-45%，CDK4/6抑制剂也进行了一系列的探索研究，并取得一定的效果，将来有可能成为新的治疗手段。本文重点讨论CDK4/6抑制剂在NSCLC的研究进展，包括作用机制、获批药物、在NSCLC中的临床研究进展、疗效预测生物标记物及局限性等。

## Abstract (en)

Lung cancer is the leading cause of cancer-related death worldwide. Non-small cell lung cancer (NSCLC) accounts for 80%-85% of lung cancer. Malignant tumors can proliferate indefinitely, and cell cycle regulation is abnormal which is one of the characteristics of malignant tumor. Cyclin D-dependent kinase (CDK) 4/6 inhibitor can prevent tumor cells from entering S phase through G1 phase and inhibit tumor proliferation. The CDK4/6 inhibitor has achieved good effect in hormone receptor positive breast cancer, and CDK4/6 inhibitor combined with endocrine therapy has become a standard treatment for this kind of patients. Cell cycle regulation is usually abnormal in NSCLC, which incidence is about 22%-45%. CDK4/6 inhibitors have also carried out a series of research and achieved some results, which may become a new treatment in the future. This review focuses on the research progress of CDK4/6 inhibitors in NSCLC, including the mechanism and the approved drugs, clinical research progress in NSCLC, biomarkers for efficacy prediction and current limitations.

肺癌是严重威胁人类健康的疾病，无论在中国还是全世界，其均为发病率、死亡率位居第一的恶性肿瘤^[1, 2]^。其中非小细胞肺癌（non-small cell lung cancer, NSCLC）占80%-85%，并且很大一部分患者发现时已经为晚期，失去手术根治的机会。近年来，靶向驱动基因表皮生长因子受体（epidermal growth factor receptor, *EGFR*）、间变性淋巴瘤激酶（anaplastic lymphoma kinase, *ALK*）的靶向治疗取得了非常好的效果^[3, 4]^，与化疗相比，明显延长了无进展生存期（progression-free survival, PFS）和总生存期（overall survival, OS），但是，靶向治疗不可避免地会产生耐药；以PD-1和PD-L1为代表的免疫治疗^[5]^取得了长足进步，但是，整体获益人群有限，需要积极寻找新的治疗靶点。

细胞周期通路的调控异常在NSCLC中非常常见^[6-8]^，有22%-25%患者存在细胞周期蛋白D1-3（cyclin D1-3）和细胞周期依赖激酶（cyclin D-dependence kinase, CDK）4/6扩增、点突变等，导致细胞增殖能力过度活化；6%-45%的患者有CDK抑制因子（CKIs，又称INK4家族）CDKN2A（p16^INK4a^）、CDKN2B（p15^INK4b^）、CDKN2C（p18^INK4c^）和CDKN2D（p19^INK4d^）的缺失、点突变和多重改变^[8, 9]^，导致抑制细胞增殖能力减弱，为CDK4/6抑制剂的治疗提供理论依据。临床上，CDK4/6抑制剂单用或联合来曲唑、氟维司群等内分泌治疗药物在雌激素受体（estrogen receptor, ER）阳性的晚期乳腺癌治疗中取得了非常好的疗效，明显延长了患者PFS和OS^[10, 11]^。在NSCLC中，CDK4/6抑制剂也进行了大量探索性研究，主要集中在作用机制、单药、联合其他药物以及优势人群的选择等方面，现综述如下。

## 1 CDK4/6抑制剂作用机制及目前获批药物

多种细胞外信号通过CDK4/6桥梁作用来影响细胞周期进程^[12]^。在正常细胞中，促进生长的信号，通过包括有丝分裂原受体、RAS、RAF、MAPK、Fun、Jun通路和PI3K、PIP3、AKT/PKB、β-Catinin等多种通路激活Cyclin D，进一步和CDK4/6结合启动视网膜母细胞瘤蛋白（retinoblastoma protein, Rb）初始磷酸化，进而激活CyclinE-CDK2促使Rb高度磷酸化形成正反馈，使Rb和转录因子E2F分离，促使细胞通过R点，进入细胞增殖分裂期^[13]^。正常情况下，Cyclin D-CDK4/6复合物和P16（CDKN2A）、P15（CDKN2B）结合，抑制CyclinD-CDK4/6对Rb的初始磷酸化，发挥抑制细胞增殖的作用^[12, 14]^。

在肿瘤细胞中，由于Cyclin D1过表达、INK4（CDKN2A、CDKN2B等）失活、*CDK4/6*基因突变等细胞增殖环路失去正常调控，CDK4/6抑制剂可以和CDK4/6结合抑制Cyclin D-CDK4/6功能，阻滞肿瘤细胞通过R点，发挥抗肿瘤的机制^[14]^。尽管Rb是CDK4/6在细胞周期调控中的主要靶标，但CDK4/6抑制剂同时也影响细胞分化、凋亡激活、线粒体活性、调节免疫、促进衰老以及细胞代谢等许多非Rb靶点功能^[15-17]^。然而，在机体内CDK4/6抑制剂究竟如何发挥抗肿瘤作用，仍不是非常清楚，因此，没有找到准确预测其疗效的生物标记物。

目前，在世界范围内批准上市的CDK4/6抑制剂主要有3种：哌柏西利（Palbociclib）、瑞柏西利（Ribociclib）和安博西利（Abemaciclib）。哌柏西利在2016年最早批准上市，在结构式上，瑞柏西利和哌柏西利非常类似。体外研究显示安博西利和瑞柏西利对CDK4的抑制作用要强于CDK6，而哌柏西利对于CDK4和CDK6的抑制作用类似^[18]^。3种药物均为口服，哌柏西利和瑞柏西利半衰期分别为28 h和30 h-50 h，用法分别为每日125 mg和600 mg，口服1次，连用3周，停用1周，主要剂量限制性毒性为中性粒细胞减少；安博西利半衰期为21 h，每次200 mg，每日两次，持续口服，主要剂量限制性毒性为消化道反应^[19]^。

## 2 CDK4/6抑制剂在NSCLC中的临床研究进展

### 2.1 CDK4/6抑制剂单药治疗

#### 2.1.1 哌柏西利（Palbociclib）

哌柏西利在NSCLC中的研究多为Ⅰ期/Ⅱ期研究。在晚期肺鳞癌Lung-MAP（SWOG S1400）试验中一组队列为哌柏西利对比多西他赛，这些患者均为CDK4/6突变或Cyclin D扩增；2018年美国临床肿瘤学协会（American Society of Clinical Oncology, ASCO）年会公布结果：32例患者接受哌柏西利治疗，有2例患者部分缓解（partial response, PR）（均为Cyclin D扩增）、12例患者疾病稳定（stable disease, SD），客观有效率（objective response rate, ORR）为6.3%，疾病控制率为44.0%^[20]^。另外一项哌柏西利Ⅱ期研究^[21]^纳入患者为肿瘤组织免疫组化p16缺失、一线治疗失败后的患者，在19例接受哌柏西利治疗的患者中，16例进行疗效评估，SD患者8例，疾病控制率为50.0%，客观有效率为0.0%，中位PFS为3.2个月（95%CI：2.1个月-6.0个月），中位OS为7.7个月（95%CI：4.0个月-13.5个月）；在SD患者中，中位PFS长达6.1个月，中位OS长达16.5个月，均较疾病进展患者中位PFS（2.2个月）和中位OS（4.2个月）明显延长（*P* < 0.001和*P*=0.003）。该研究提示，在CDK4/6抑制剂临床终点选择上，PFS、OS终点优于ORR的终点。

#### 2.1.2 安博西利（Abemeciclib）

安博西利在晚期NSCLC中也进行了一系列研究^[22-25]^。在Ⅰ期针对晚期NSCLC的研究中^[22]^，纳入患者均为常规治疗失败的NSCLC，既往接受1线-10线治疗，中位接受治疗线数为4线；入组68例患者中，疾病控制率为49%（33/68），6个月PFS率为26%，4例患者PFS超过12个月；2例患者达到PR，ORR为2.9%。进一步分析2例PR的患者，1例患者为*Kras*突变，1例为CDKN2A拷贝数缺失的肺鳞癌患者。亚组分析，*Kras*突变29例患者，疾病控制率为55%（16/29），高于K-ras野生型患者的39%（13/33）；在SD时间超过24周的患者中，*Kras*突变患者也明显高于*Kras*野生型患者（31% *vs* 12%）；*Kras*突变型患者中位PFS较*Kras*野生型患者延长（2.8个月*vs* 1.9个月）。

基于Ⅰ期研究在NSCLC中良好的效果，安博西利开展JUNIPER研究^[6, 23, 25, 26]^。该研究纳入患者为*Kras*突变患者，随机按3:2分组，安博西利+最佳支持治疗对比厄洛替尼+最佳支持治疗，在既往接受过多线治疗的Ⅳ期NSCLC患者中进行。患者被随机分为安博西利200 mg *q12h*组或厄洛替尼150 mg *qd*组。共入组453例患者，其中安博西利组270例，厄洛替尼组183例，主要研究终点为OS，次要研究终点为PFS和ORR。结果显示：与厄洛替尼组相比，安博西利组中位PFS明显延长（3.6个月*vs* 1.9个月，*P* < 0.001）、ORR（8.9% *vs* 2.7%, *P*=0.01）和疾病控制率（54.4% *vs* 31.7%）均明显提高；但两组间OS并无显著差异（7.4个月*vs* 7.8个月，*P*=0.771），该研究没有达到主要临床终点，宣告失败。

另外一项Ⅱ期随机研究^[24]^入组为一线铂类为基础化疗失败的晚期肺鳞癌，比较安博西利和多西他赛的疗效。入组159例，按2:1随机分配，安博西利组106例，多西他赛组53例。两组患者中位PFS分别为2.5个月和4.2个月（HR=1.77, 95%CI: 1.17-2.67, *P*=0.006, 8），中位OS分别为7.0个月和12.4个月（HR=1.33, 95%CI: 0.88-2.02, *P*=0.174, 6）；疾病控制率分别为50.9%和64.2%，ORR分别为2.8%和20.8%。因此，和多西他赛相比，单药安博西利没有改善晚期肺鳞癌患者的二线治疗生存^[24]^。

综上所述，单药CDK4/6抑制剂尽管在晚期NSCLC中有一定的疗效，但是整体疗效较差，需要探索更好的治疗策略。

### 2.2 CDK4/6抑制剂联合其他药物

#### 2.2.1 CDK4/6抑制剂联合MEK抑制剂

早期研究结果表明，*ras*突变可以激活Cyclin D，促进细胞增殖，并且在结直肠癌和胰腺癌临床前研究模型中，证实CDK4/6抑制剂和MEK抑制剂具有协同增效作用，因此，该策略也在肺癌治疗中引起极大的兴趣^[27, 28]^。在*Kras*突变的肺癌细胞系中，MEK抑制剂曲美替尼（Tremetinib）联合哌柏西利可以明显降低细胞存活率；在对MEK抑制剂耐药细胞中，部分是因为*P16*突变导致，联合CDK4/6抑制剂能增加细胞敏感性^[29]^。目前正在开展一项MEK抑制剂比美替尼（Binimetinib）联合哌柏西利在*Kras*突变NSCLC的Ⅰ期/Ⅱ期研究（NCT03170206）。

#### 2.2.2 CDK4/6抑制剂联合PI3KCA抑制剂治疗*PI3KCA*突变肺鳞癌

在肺鳞癌中，PI3K通路异常是非常重要的一种亚型，通常表现为*PIK3CA*突变、扩增和*PTEN*缺失，在肺鳞癌中其发生率分别为10%-15%、50%和20%-30%^[25]^。PI3K是CDK4/6的上游活化信号，通过Cyclin D的活化发挥作用^[30]^。临床前肺鳞癌患者人源肿瘤异种移植（patient-derived tumor xenograft, PDX）模型显示，在*PI3KCA*突变的模型中，CDKN2A纯合性缺失和杂合性缺失比例非常高，单用PI3K抑制剂BKM1220或者BYL719在动物体内有效率为33%，主要是针对*PI3K*突变患者。联合PI3K抑制剂和安博西利，在PI3K突变肺鳞癌动物模型中，较单药PI3K抑制剂更有效^[31]^。CDK4/6抑制剂（哌柏西利或安博西利）联合PI3K抑制剂，能明显抑制模型中肿瘤细胞的生长，并且耐受良好。在4例裸鼠中，有3例出现不同程度的肿瘤缩小^[31]^。可能的机制在于PI3K突变通过Akt、mTOR会导致Cyclin D激活，同时P16缺失减弱对CDK4/6抑制作用，联合PI3K和CDK4/6抑制剂可能同时减少Cyclin D1和增加对CDK4/6的抑制，起到协同作用^[31]^。目前正在开展一项PI3K/mTOR抑制剂（Gedatolisib）联合哌柏西利在晚期实体肿瘤中的临床研究（NCT03065062）。

#### 2.2.3 CDK4/6抑制剂联合mTOR抑制剂

在基础研究中，CDK4/6抑制剂能对多种肺癌细胞系H520、H538、H2347和H125发挥抑制作用，在联合mTOR抑制剂能协同增效并且逆转对CDK4/6抑制剂耐药^[21, 32]^。其机制可能是：CDK4/6抑制剂可代偿性使Cyclin D1增加，这种增加反馈性影响AKT/mTOR通路，导致磷酸化P70S6K的下调，而mTOR抑制剂能阻断CDK4/6抑制剂治疗后Cyclin D1增加，发挥协同作用^[21]^。

#### 2.2.4 CDK4/6抑制剂联合细胞毒性化疗药物

CDK4/6抑制剂和化疗药物可能有拮抗作用，因此，需要注意用药顺序。在Calu-6细胞系异种移植裸鼠模型研究中发现，CDK4/6抑制剂联合吉西他滨增强抗肿瘤活性，但是没有出现G_1_细胞周期停滞，可能与核苷还原酶表达的降低有关^[33]^。目前正在开展一项吉西他滨联合安博西利在晚期NSCLC中的Ⅰ期研究（NCT02079636）。

#### 2.2.5 CDK4/6抑制剂联合PD-1和PD-L1抑制剂

CDK4不但能促进肿瘤细胞增殖，同时也是肿瘤微环境成熟的重要条件，能影响肿瘤细胞抗原呈递和表达^[34]^。CDK4/6能够促进T细胞PD-1表达，增加T细胞浸润，因此，哌柏西利联合PD-1抑制剂能增强肿瘤退缩，改善人源肿瘤异种移植模型裸鼠的生存时间^[35, 36]^。目前一项帕博利珠单抗（Pembrolizumab）联合安博西利治疗晚期NSCLC的研究正在进行（NCT02079636）。

综上所述，CDK4/6抑制剂和多种药物联合使用可能协同增效，但是这些研究多数来自临床前数据，需要进一步得到临床验证。

## 3 CDK4/6抑制剂在NSCLC中疗效预测生物标记物

### 3.1 *Kras*突变

在*Kras*突变的肺癌细胞系中，CDK4的敲除会导致肺癌细胞生长明显抑制。同时对于*Kras*突变细胞在移植裸鼠模型中研究^[37]^发现，应用CDK4/6抑制剂哌柏西利能明显降低转移率（17% *vs* 75%），减缓肿瘤生长。在安博西利的NSCLC Ⅰ期临床研究中发现，*Kras*突变患者疾病控制率明显高于*Kras*野生型（55% *vs* 29%），SD > 24周者，*Kras*突变型优于野生型（31% *vs* 12%），中位PFS也延长（2.8个月*vs* 1.9个月）^[22]^。这些结果提示，*Kras*突变可能是CDK4/6抑制剂的有效人群，然而，在JUNIPER研究中，*Kras*突变型患者的ORR仅为8.9%，PFS为2.7个月^[25]^，远较*EGFR*突变患者应用EGFR-TKI疗效差，因此，*Kras*突变并不是很好的生物标记物。

### 3.2 *Kras*和*LKB1*共突变

在安博西利NSCLC Ⅰ期研究中，进一步分析发现，在4例*Kras*突变合并有*LKB1*突变的患者，均取得肿瘤的不同程度缩小，其中2例接近PR^[22]^。因此，是否对于*Kras*和*LKB1*共突变的NSCLC患者能获益更多，有待进一步临床验证。

### 3.3 P16（CDKN2A）缺失（免疫组化阴性）

CDKN2A作为CDK4/6抑制因子，在正常细胞周期中对于控制Cyclin D介导CDK4/6激活起到非常重要作用，而CDKN2A在NSCLC中经常发生缺失，因此，CDKN2A可能是潜在生物标记物。在一项应用CDKN2A阴性作为筛选标志的Ⅱ期晚期NSCLC研究^[21]^中，CDKN2A阴性患者接受哌柏西利治疗的16例患者中，并没有出现ORR的患者，PFS仅为2.2个月，因此CDKN2A阴性并不是理想的筛选生物标记物。

### 3.4 Rb表达功能完整

Cyclin D和CDK4/6结合后逐步磷酸化Rb，使其丧失对细胞周期的调控，细胞通过R点，进入增殖。因此，对于Rb存在突变、缺失的患者可能不能从CDK4/6抑制剂治疗中获益。一项基础研究^[38]^显示：CDK4/6抑制剂能诱导Rb蛋白表达正常的肺癌细胞凋亡，其机制主要通过下调凋亡抑制物（FOXM1和Survivin），同时上调促凋亡因子SMAC和细胞色素C表达，促进肿瘤凋亡发挥抗肿瘤作用；而在Rb缺失突变患者中，CDK4/6抑制剂无促进肿瘤凋亡的功能。因此CDK4/6抑制剂单独应用在*Rb*突变的患者中可能无效。小细胞肺癌90%患者存在Rb的改变^[39]^，而在NSCLC中90%患者的Rb功能正常^[6]^，因此从信号通路角度提示，CDK4/6抑制剂在肺癌领域的研究主要集中于NSCLC，一旦发生*Rb*突变可能提示CDK4/6抑制剂无效。

### 3.5 *SMARCA4*缺失

*SMARCA4*基因是抑癌基因，该基因编码的蛋白是Swi/SNF蛋白家族的一员，具有螺旋酶和ATP酶活性，能通过改变基因周围的染色质结构来调节多种基因的转录^[39, 40]^。既往研究发现，*SMARCA*突变是卵巢高钙血型小细胞癌（small cell carcinoma of the ovary of hypercalcemic type, SCCOHT）的驱动基因^[41]^，该基因的缺失导致Cyclin D下降引起对CDK4/6抑制剂敏感性增加，CDK4/6抑制剂治疗有效^[42]^。在NSCLC中*SMARCA4*突变失活，大约有10%左右，也存在这种机制^[43]^。在*SMARCA4*缺失的情况下，SMARCA2影响SWI/SNF的亚基，可以调节Cyclin D和对药物的敏感性。*SMARCA4/2*缺失一方面通过限制Cyclin D1染色质的可及性，另一方面抑制Cyclin D1关键的转录激活因子c-jun来发挥效果。在NSCLC体外细胞系和小鼠体内证实*SMARCA4*缺失和CDK4/6抑制剂合成致死作用^[43]^。在临床患者中发现*SMARCA4*缺失患者存在Cyclin D表达下降，这类患者可能预后较差，并且，其中20%患者存在*Kras*突变。因此，*SMARCA4*突变患者可能是CDK4/6获益的有效指标。

## 4 局限性

CDK4/6抑制剂尽管在NSCLC中进行了一系列的研究，主要集中在Ⅰ期、Ⅱ期研究，但是在NSCLC中的整体疗效没有达到预期效果，和激素受体阳性的乳腺癌相比差距较大。未来研究主要集中在CDK4/6抑制剂联合其他药物治疗，希望将来在NSCLC中可以获得比较理想的效果。

综上所述，CDK4/6抑制剂在晚期NSCLC治疗中可能有一席之地。未来，希望有更多基础和临床研究继续探讨CDK4/6抑制剂的精准获益人群，以期获得较好的临床效果；同时，从信号通路、调节肿瘤免疫微环境等多个角度制定联合治疗策略，以期取得最佳疗效。

## References

[1] Chen W, Zheng R, Baade PD (2016). Cancer statistics in China, 2015. CA Cancer J Clin.

[2] Bray F, Ferlay J, Soerjomataram I (2018). Global cancer statistics 2018: GLOBOCAN estimates of incidence and mortality worldwide for 36 cancers in 185 countries. CA Cancer J Clin.

[3] Zhang T, Wan B, Zhao Y (2019). Treatment of uncommon EGFR mutations in non-small cell lung cancer: new evidence and treatment. Transl Lung Cancer Res.

[4] Thai AA, Solomon BJ (2018). Treatment of ALK-positive nonsmall cell lung cancer: recent advances. Curr Opin Oncol.

[5] Almutairi AR, Alkhatib N, Martin J (2019). Comparative efficacy and safety of immunotherapies targeting the PD-1/PD-L1 pathway for previously treated advanced non-small cell lung cancer: A Bayesian network *meta*-analysis. Crit Rev Oncol Hematol.

[6] Cancer Genome Atlas Research Network (2012). Comprehensive genomic characterization of squamous cell lung cancers. Nature.

[7] Sherr CJ, Beach D, Shapiro GI (2016). Targeting CDK4 and CDK6: from discovery to therapy. Cancer Discov.

[8] Otto T, Sicinski P (2017). Cell cycle proteins as promising targets in cancer therapy. Nat Rev Cancer.

[9] Vanarsdale T, Boshoff C, Arndt KT (2015). Molecular pathways: targeting the Cyclin D-CDK4/6 axis for cancer treatment. Clin Cancer Res.

[10] Finn RS, Martin M, Rugo HS (2016). Palbociclib and letrozole in advanced breast cancer. N Engl J Med.

[11] Goetz MP, Toi M, Campone M (2017). MONARCH 3: abemaciclib as initial therapy for advanced breast cancer. J Clin Oncol.

[12] Sherr CJ, Roberts JM (1999). CDK inhibitors: positive and negative regulators of G1-phase progression. Genes Dev.

[13] Harbour JW, Luo RX, Dei SA (1999). Cdk phosphorylation triggers sequential intramolecular interactions that progressively block Rb functions as cells move through G1. Cell.

[14] Classon M, Harlow E (2002). The retinoblastoma tumour suppressor in development and cancer. Nat Rev Cancer.

[15] Bendris N, Lemmers B, Blanchard JM (2015). Cell cycle, cytoskeleton dynamics and beyond: the many functions of cyclins and CDK inhibitors. Cell Cycle.

[16] Lim S, Kaldis P (2013). Cdks, cyclins and CKIs: roles beyond cell cycle regulation. Development.

[17] Hydbring P, Malumbres M, Sicinski P (2016). Non-canonical functions of cell cycle cyclins and cyclin-dependent kinases. Nat Rev Mol Cell Biol.

[18] Tripathy D, Bardia A, Sellers WR (2017). Ribociclib (LEE011): mechanism of action and clinical impact of this selective cyclin-dependent kinase 4/6 inhibitor in various solid tumors. Clin Cancer Res.

[19] Klein ME, Kovatcheva M, Davis LE (2018). CDK4/6 inhibitors: the mechanism of action may not be as simple as once thought. Cancer Cell.

[20] Lam VK, Papadimitrakopoulou V (2018). Master protocols in lung cancer: experience from lung master protocol. Curr Opin Oncol.

[21] Gopalan PK, Villegas AG, Cao C (2018). CDK4/6 inhibition stabilizes disease in patients with p16-null non-small cell lung cancer and is synergistic with mTOR inhibition. Oncotarget.

[22] Patnaik A, Rosen LS, Tolaney SM (2016). Efficacy and safety of abemaciclib, an inhibitor of CDK4 and CDK6, for patients with breast cancer, non-small cell lung cancer, and other solid tumors. Cancer Discov.

[23] Goldman JW, Shi P, Reck M (2016). Treatment rationale and study design for the JUNIPER study: a randomized phase Ⅲ study of abemaciclib with best supportive care versus erlotinib with best supportive care in patients with stage Ⅳ non-small-cell lung cancer with a detectable *KRAS* mutation whose disease has progressed after platinum-based chemotherapy. Clin Lung Cancer.

[b24] 24Scagliotti GV. A randomized phase 2 study of abemaciclib versus docetaxel in patients with stage Ⅳ squamous non-small cell lung cancer (sqNSCLC) previously treated with platinum-based chemotherapy. Chicago: 2018473.

[25] Hew GA (2018). A randomized phase 3 study of abemaciclib versus erlotinib in previously treated patients with stage Ⅳ NSCLC with*KRAS* mutation: JUNIPER. J Clin Cancer.

[b26] 26Jonathan W, Goldman PSMR, Andrew Koustenis KCH. A study of abemaciclib (LY2835219) in participants with previously treated*KRAS* mutated lung cancer (JUNIPER).

[27] Franco J, Witkiewicz AK, Knudsen ES (2014). CDK4/6 inhibitors have potent activity in combination with pathway selective therapeutic agents in models of pancreatic cancer. Oncotarget.

[28] Lee MS, Helms TL, Feng N (2016). Efficacy of the combination of MEK and CDK4/6 inhibitors *in vitro* and *in vivo* in*KRAS* mutant colorectal cancer models. Oncotarget.

[29] Tao Z, Le Blanc JM, Wang C (2016). Coadministration of trametinib and palbociclib radiosensitizes*KRAS*-mutant non-small cell lung cancers *in vitro* and *in vivo*. Clin Cancer Res.

[30] Averous J, Fonseca BD, Proud CG (2008). Regulation of cyclin D1 expression by mTORC1 signaling requires eukaryotic initiation factor 4E-binding protein 1. Oncogene.

[31] Shi R, Li M, Raghavan V (2018). Targeting the CDK4/6-Rb pathway enhances response to PI3K inhibition in *PIK3CA*-mutant lung squamous cell carcinoma. Clin Cancer Res.

[32] Haines E, Chen T, Kommajosyula N (2018). Palbociclib resistance confers dependence on an FGFR-MAP kinase-mTOR-driven pathway in *KRAS*-mutant non-small cell lung cancer. Oncotarget.

[33] Gelbert LM, Cai S, Lin X (2014). Preclinical characterization of the CDK4/6 inhibitor LY2835219: *in-vivo* cell cycle-dependent/independent anti-tumor activities alone/in combination with gemcitabine. Invest New Drugs.

[34] Goel S, Decristo MJ, Watt AC (2017). CDK4/6 inhibition triggers anti-tumour immunity. Nature.

[35] Zhang J, Bu X, Wang H (2018). Cyclin D-CDK4 kinase destabilizes PD-L1 via cullin 3-SPOP to control cancer immune surveillance. Nature.

[36] Deng J, Wang ES, Jenkins RW (2018). CDK4/6 inhibition augments antitumor immunity by enhancing T-cell activation. Cancer Discov.

[37] Puyol M, Martin A, Dubus P (2010). A synthetic lethal interaction between *K-Ras* oncogenes and Cdk4 unveils a therapeutic strategy for non-small cell lung carcinoma. Cancer Cell.

[38] Thangavel C, Boopathi E, Liu Y (2018). Therapeutic challenge with a CDK 4/6 inhibitor induces an RB-dependent SMAC-mediated apoptotic response in non-small cell lung cancer. Clin Cancer Res.

[39] George J, Lim JS, Jang SJ (2015). Comprehensive genomic profiles of small cell lung cancer. Nature.

[40] Coordinator EA, Campbell JC, Data CS (2014). Comprehensive molecular profiling of lung adenocarcinoma. Nature.

[41] Jelinic P, Schlappe BA, Conlon N (2016). Concomitant loss of SMARCA2 and SMARCA4 expression in small cell carcinoma of the ovary, hypercalcemic type. Mod Pathol.

[42] Xue Y, Meehan B, Macdonald E (2019). CDK4/6 inhibitors target SMARCA4-determined cyclin D1 deficiency in hypercalcemic small cell carcinoma of the ovary. Nat Commun.

[43] Xue Y, Meehan B, Fu Z (2019). SMARCA4 loss is synthetic lethal with CDK4/6 inhibition in non-small cell lung cancer. Nat Commun.

